# Comparative study of natural rubber and styrene-butadiene rubber blends reinforced with different carbon black grades for tire tread production

**DOI:** 10.1038/s41598-026-65435-2

**Published:** 2026-08-11

**Authors:** R. Singer, A. M. Ollick, M. Elhadary, Ibrahim M. El-Sherbiny, A. Gomma

**Affiliations:** 1 Department of Mechanical Engineering, Faculty of Engineering, Alexandria University, Alexandria, Egypt; 2 Center for Materials Science (CMS), Zewail City of Science and Technology, 6th of October, Giza Egypt

**Keywords:** NR/SBR blends, Carbon black, Tire tread compounds, Mechanical properties, Chemistry, Engineering, Materials science

## Abstract

In this study, the effect of carbon black (CB) on the properties of natural rubber (NR) and styrene–butadiene rubber (SBR) blends were investigated. Carbon black with different grades, N330 and N550, was used as a reinforcing filler of NR/SBR blends. The rubber compounds were prepared using a two-roll mill followed by sulfur vulcanization at a fixed filler content of 50 phr and different NR/SBR blend ratios. The mechanical properties were estimated through tensile strength, tensile modulus, elongation at break, toughness, tear strength, and hardness measurements. By analyzing the results, it was found that both the NR/SBR blend ratio and carbon black types significantly affect the mechanical characteristics of the NR/SBR compounds. NR/SBR blends reinforced with CB N330 exhibited higher strength, while compounds reinforced with CB N550 showed lower strength. This behavior is due to the smaller particle size and higher surface area of CB N330, which improves filler–rubber interactions and reinforcing efficiency. Moreover, the NR/SBR blend (75:25) reinforced with CB N330 showed higher tensile strength, modulus, elongation at break, toughness, tear strength, and hardness than the corresponding N550-filled compound, with an increase of 17.53%, 5.5%, 13.42%, 43.29%, 46.3%, and 22.2%, respectively. Additionally, CB N330-filled NR/SBR compounds showed higher residual mass compared to CB N550-filled blends, indicating stronger filler–polymer interactions. Overall, the results explain the relationship between carbon black properties and NR/SBR blend formulation, providing practical guidance for optimizing carbon black selection in tire tread compounds that require enhanced mechanical strength, fracture resistance, and thermal stability.

## Introduction

Rubber is a versatile material that is widely used in applications such as adhesives, footwear, automotive, civil, and electrical components, as well as hoses, tubes, and various industrial products. Among these applications, rubber plays a vital role in the tire industry. Natural rubber (NR) is one of the most widely used elastomers due to its renewable nature and excellent mechanical properties, including high tensile strength, large elongation at break, and superior tear resistance compared to most synthetic rubbers. These properties are mainly attributed to its ability to undergo strain-induced crystallization and its high resilience, which make NR suitable for demanding applications such as tires, conveyor belts, sealing systems, vibration-damping components, and molded rubber products^1–4^.

NR is extensively used in tire manufacturing, especially for heavy trucks and aircraft, where low heat build-up is essential. While passenger car tire treads are generally based on synthetic rubbers, heavy-duty truck tires contain 50–100% NR, and aircraft tires are almost entirely NR. In addition, several tire components, including body compounds, sidewalls, and bead fillers, are also made from NR because of its good flex fatigue and favorable mechanical properties^5^.

Styrene-butadiene rubber (SBR), a non-polar synthetic rubber, is produced by the copolymerization of styrene and butadiene. SBR is generally used in wear-resistant applications due to its good resistance to oil, heat, hydrocarbon solvents, water, and steam, as well as favorable mechanical properties. It has good abrasion resistance and thermal aging properties. In tire applications, SBR is particularly valued for its compatibility with reinforcing fillers, improved abrasion resistance, enhanced traction, reduced rolling resistance, and resistance to crack initiation^6–8^. Thus, SBR has been used in various products such as tire sidewalls, belts, hoses, footwear, foamed materials, and related tire products^9,10^.

In recent years, considerable research efforts have focused on polymer blending as an effective strategy for enhancing the physical properties of the final vulcanized materials^11^. For the same reason, rubber blends are commonly used in the rubber industry to obtain the required product performance and the best combination of compound properties, processability, and cost^12,13^.

The most commonly used rubber blends in the tire industry are NR/butadiene rubber (NR/BR) and NR/styrene–butadiene rubber (NR/SBR)^12,14^. Blending natural rubber (NR) with synthetic rubber such as styrene-butadiene rubber (SBR) is intended to improve the mechanical properties of NR, such as hardness and abrasion resistance, especially when the blend is used as a basic material for tires. Conversely, SBR is usually blended with NR to improve its tensile and tear properties since SBR has better resistance to crack initiation, abrasion resistance, lower heat-up performance, and high filler-content capacity^15,16^. However, compatibility between NR and SBR is limited without the addition of other ingredients acting as compatibilizers or reinforcing agents, which improve interfacial adhesion and overall blend homogeneity^17^.

Several studies have addressed the blends of natural rubber (NR) and various synthetic rubbers. Some studies have investigated the decomposition of various types of tires in the pyrolysis reaction, elucidating how the rubber composition affects the final products^18–20^. Additionally, the ratio of natural to synthetic rubber significantly affects the properties of the final composition^21–25^.

Wirjosentono et al.^26^ studied the mechanical and morphological properties of natural rubber–styrene–butadiene rubber (NR/SBR) blends reinforced with natural microbentonite (NMB). The tensile strength of NR/SBR blends at a constant blend ratio of 50/50 increased with increasing NMB loading up to 3 phr; however, a further increase in NMB content up to 7 phr resulted in a reduction in tensile strength. In contrast, the elongation at break decreased continuously with increasing NMB content, while Young’s modulus increased steadily from 0.023 to 0.041 MPa as the NMB loading increased up to 7 phr.

Bondan^27^ investigated the tensile properties and morphological characteristics of different NR-based composites designed for solid forklift tires. The results indicated that the formulation containing 60% NR and 20% SBR showed balanced mechanical properties, with a hardness of 78 Shore A, tensile strength of 17.3 MPa, tear strength of 55.2 kN/m, abrasion resistance of 160.8 mm³, and a modulus at 300% elongation of 6.6 MPa.

On an industrial scale, rubber blending is carried out using a two-roll mill machine, where the rubber matrices and all ingredients are in a solid state at low temperature to minimize chemical interactions between the rubber matrices and blending ingredients. Consequently, rubber blend compatibility can be reached by intensive mechanical mixing procedures^28^. Mansila et al. compared the compatibility of natural rubber/styrene-butadiene rubber (NR/SBR) blends prepared using a two-roll mill machine and via solution blending^29^. The results indicated that no significant difference in the degree of molecular entanglement was observed between the two methods.

Furthermore, rubber reinforcement is a critical factor in tire performance, as the incorporation of fillers enhances key properties while reducing compound costs. Fillers are commonly used to improve mechanical properties such as tensile modulus, strength, resilience, abrasion resistance, wet traction, and rolling resistance^30^. Solid fillers, including carbon black, silica, and clay, can also act as compatibilizing agents in rubber blends, particularly during solid-state mixing using a two-roll mill. Carbon black remains the most widely used reinforcing filler in tire compounds, especially grade N330 in truck tire applications. Decreasing carbon black particle size and increasing surface area generally enhance mechanical properties at a given loading; however, finer grades may also increase heat build-up, leading to trade-offs between reinforcement and dynamic performance^31^.

Gunawan et al.^31^ studied the NR formulation for military vehicle tread using different carbon black grades (N220/N550) as reinforcing fillers. The physical and mechanical properties of the specimens, including curing time, tensile strength, tear strength, elongation at break, and compression, were evaluated. The results showed that the RSS 1/CB N220 compound exhibited significantly higher strength and stiffness.

Chollakup et al.^32^ investigated the mechanical characteristics and energy dissipation of carbon black/rubber compounds. Carbon black with different grades, N330 and N220, was used as a reinforcing agent for natural rubber. In addition, the effects of different loadings of carbon black N330 and N220 at 40, 45, 50, and 55 phr were determined. The results indicated that the strength of rubber composites increased as the carbon black content increased. Additionally, at the same bound rubber level, rubber composites with N220 presented lower dissipation energy, reduced heat build-up, and better mechanical properties than those with N330.

Kherbouche et al.^33^ investigated the rheological properties and mechanical strength characteristics of natural rubber vulcanizates by varying the type and content of carbon black. For N220-reinforced NR, the addition of filler resulted in improved tensile strength up to a loading of 70 phr. However, with respect to elongation at break, a reduction in elasticity was observed. Nevertheless, the N220-filled rubber compounds exhibited slightly higher hardness values than those reinforced with N550.

Despite these contributions, a critical gap remains in the literature. Systematic comparisons between carbon black grades N330 and N550 in NR/SBR blends, particularly under constant filler loading, identical vulcanization and processing conditions, and across a wide range of NR/SBR blend ratios, remain limited. More importantly, the combined effect of carbon black grade and blend composition on the mechanical, thermal, swelling, and network characteristics of NR/SBR blends has not been comprehensively established.

Accordingly, this work provides a systematic and controlled investigation of NR/SBR blends based on RSS 3 natural rubber and SBR 1502 reinforced with N330 and N550 carbon black at fixed filler loading. All compounds were prepared using the same sulfur vulcanization system, two-roll mill mixing procedure, compression molding pressure, vulcanization temperature, and curing conditions to ensure reliable comparison between formulations. The effects of blend ratio and filler grade on the mechanical, thermal, swelling, and apparent crosslink density behavior were evaluated in detail. By correlating filler characteristics with blend composition under well-defined manufacturing and vulcanization conditions, this study provides clearer insight into the reinforcement behavior of NR/SBR blends and offers practical guidelines for optimizing tire tread compositions.

## Experimental

### Materials

Natural rubber (NR), in the form of Ribbed Smoked Sheets (RSS 3), was supplied by Van Xuan Industries Company Limited (Ho Chi Minh City, Vietnam). Styrene–butadiene rubber (SBR 1502), commercially designated as HIPREN EM 1502T, was supplied by HIP-Petrohemija LLC (Pančevo, Serbia). N-cyclohexyl-2-benzothiazole sulfenamide (CBS) was used as an accelerator, while N-(1,3-dimethylbutyl)-N′-phenyl-p-phenylenediamine (6PPD) and 2,2,4-trimethyl-1,2-dihydroquinoline polymer (TMQ) were employed as antioxidants. Zinc oxide (ZnO) and insoluble sulfur (S) were supplied by Elkim Chemicals (Istanbul, Turkey). Stearic acid (SA) was obtained from Sciencelab.com. Two grades of carbon black, N330 and N550, supplied by ATDM Co. LLC (Çankaya, Ankara, Turkey), were used as reinforcing fillers. N330 exhibited higher specific surface area characteristics (NSA = 78 m²/g; STSA = 75 m²/g) compared with N550 (NSA = 40 m²/g; STSA = 39 m²/g). CB N550 exhibited a higher oil absorption number (OAN = 121 × 10⁻⁵ m³/kg) than CB N330 (OAN = 102 × 10⁻⁵ m³/kg), indicating differences in aggregate structure and porosity. The bulk density values were 380 and 360 g/L for N330 and N550, respectively^34^. All materials were used as received without further purification. The formulations of the NR/SBR blends are summarized in Table 1, where the compositions are expressed in parts per hundred rubber (phr).

**Table 1:** Formulation of NR/SBR blends (phr) reinforced with carbon black N330 (A-series) and N550 (B-series).

Components	A1	A2	A3	A4	A5	B1	B2	B3	B4	B5
Natural rubber (RSS 3)	100	75	50	25	0	100	75	50	25	0
Styrene–butadiene rubber (SBR 1502)	0	25	50	75	100	0	25	50	75	100
Zinc oxide (ZnO)	5	5	5	5	5	5	5	5	5	5
Stearic acid (SA)	3	3	3	3	3	3	3	3	3	3
CB N330	50	50	50	50	50	–	–	–	–	–
CB N550	–	–	–	–	–	50	50	50	50	50
6PPD	1.5	1.5	1.5	1.5	1.5	1.5	1.5	1.5	1.5	1.5
TMQ	1.5	1.5	1.5	1.5	1.5	1.5	1.5	1.5	1.5	1.5
CBS	1.5	1.5	1.5	1.5	1.5	1.5	1.5	1.5	1.5	1.5
Sulfur	2.5	2.5	2.5	2.5	2.5	2.5	2.5	2.5	2.5	2.5

### Compounding

**Fig. 1:**
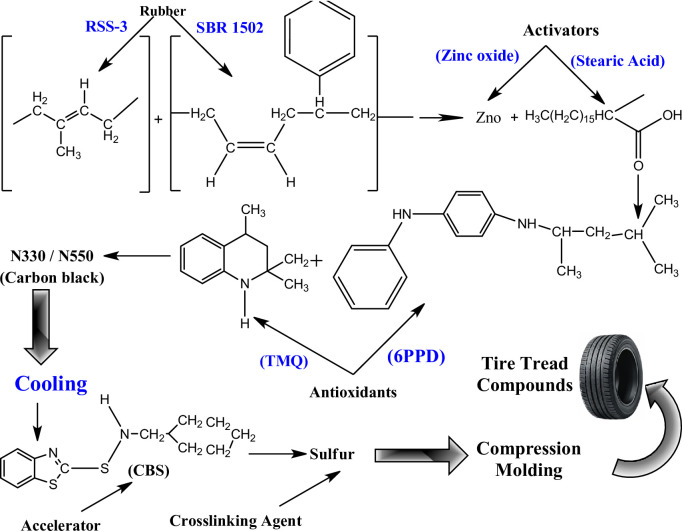
Schematic illustration of the NR/SBR tire tread formulation showing the principal rubber components (RSS-3 and SBR 1502), reinforcing fillers (carbon black N330/N550), activators (ZnO and stearic acid), antioxidants (TMQ and 6PPD), accelerator (CBS), and sulfur curing system used during compounding and vulcanization.

Natural rubber (NR) and styrene–butadiene rubber (SBR) compounds were prepared using a two-step process consisting of masterbatch preparation, followed by the addition of curatives on a laboratory two-roll mill, in accordance with ASTM D3182. Mixing was carried out at a gear ratio of 1:1.4, with roll temperatures of 50/65°C. The detailed mixing protocol used for the preparation of NR/SBR compounds is summarized in Table 2. A schematic representation of the compounding and vulcanization process, explaining the sequence of ingredient incorporation and the role of each component in the NR/SBR blends, is presented in Fig. 1.

According to the ASTM^35^ standard, the vulcanization was performed by compression molding using a hydraulic hot press at a pressure of 100 bar and a temperature of 160 ± 5 °C for 15 min. After curing, the samples were cooled by water circulation for 5 min to obtain the final vulcanized products.

**Table 2:** Two-roll mill mixing procedure for NR/SBR blends.

Stage	Step	Time (min)	Procedure
Stage 1: Masterbatch Mixing	1	0	The two-roll mill was set to front/rear roll speeds of 25/35 rpm, roll temperatures of 50/65°C, and a nip gap of 1.2 mm. Natural rubber (RSS) and SBR were first introduced into the mill for mastication and homogenization.
	2	0–3	The rubber blend was banded, and crosscutting was performed at 30-second intervals to ensure uniform filler dispersion.
	3	3–6	Zinc oxide and stearic acid were added sequentially.
	4	6–8	Antioxidants (6PPD and TMQ) were incorporated.
	5	8–12	Carbon black was gradually incorporated into the compound.
	6	12–14	Crosscutting was continued to ensure uniform dispersion.
	7	14	The compound was sheeted off at a nip gap of 0.8 mm.
	8	—	The master batch was left to cool at room temperature for 2 h.
Stage 2: Final Mixing	9	0	The cooled masterbatch was re-banded on the two-roll mill.
	10	0–2	The accelerator (CBS) was added.
	11	2–4	Sulfur was incorporated into the compound.
	12	4–5	The final sheet-off was performed at a nip gap of 0.8 mm.

### Equilibrium swelling test

An equilibrium swelling test was carried out to determine the swelling properties and estimate the apparent crosslink density of the NR/SBR rubber composites. NR/SBR specimens with dimensions of 10 × 10 mm were weighed using an analytical balance with an accuracy of 0.0001 g. NR/SBR specimens were immersed in toluene and kept in the dark for 72 h at room temperature. The reported results represent the average of at least three specimens. The swollen samples were gently wiped with filter paper to remove excess solvent from the surface and immediately weighed. The swollen specimens were subsequently dried in an oven at 60 °C until a constant weight was achieved. The swelling ratio (ζ%) of the composites was calculated as follows in Eq. (1):

1 $$\:{\upzeta\:}\mathrm{\%}\:=\left(\frac{{W}_{s}-{W}_{i}}{{W}_{i}}\right)\times\:100$$

where W_i_ is the initial (or dry) weight of the sample (mg), and W_s_ is the weight of the swollen sample (mg).

The crosslink density ($\:{{\upnu\:}}_{e}$) was estimated based on the Flory–Rehner Eq. (2)^36^:

2 $$\:{{\upnu\:}}_{e}=-\frac{\mathrm{ln}\left(1-\mathrm{V}\mathrm{r}\text{}\right)+Vr+{\upchi\:}{\mathrm{V}\mathrm{r}\text{}}^{2}}{2{\uprho\:}{V}_{s}\left({{V}_{r}}^{\frac{1}{3}}-0.5\mathrm{V}\mathrm{r}\text{}\right)}\:\:\:\:$$

where χ is the polymer–solvent interaction parameter (here equal to 0.393 for the NR/SBR-toluene system^37^, V_s_ is the molar volume of toluene(106.2 cm^3^/mol), and Vr is the volume fraction of the swollen rubber that can be calculated from Eq. (3).

3 $$\:{V}_{r}=\frac{\frac{{w}_{d}}{{\rho\:}_{r}}}{\frac{{w}_{d}}{{\rho\:}_{r}}+\frac{{w}_{s\:\:\:}-{w}_{d}}{{\rho\:}_{s}}}$$

where W_d_ is the deswollen weight, W_s_ is the swollen weight, ρ_r_ is the density of rubber blend, and ρ_s_ is the density of the solvent.

### Tensile test

Tensile properties were determined using a multiTest 5-Xt testing machine (MECMESIN) equipped with a 5 KN load cell. Tests were performed according to ASTM D412 (Type B dumbbell specimens)^38^ at a crosshead speed of 500 mm/min under controlled laboratory conditions (temperature, 24 ± 1 °C; relative humidity, 50 ± 5%). The tensile strength, elongation at break, and tensile modulus were determined from the stress–strain curves. The tensile modulus was calculated as the slope of the stress–strain curve in the initial linear (elastic) region, while the toughness was obtained from the total area under the stress-strain curve^39^. For each formulation, three specimens were tested, and the reported values represent the average of the measurements.

### Hardness test

The hardness of the rubber compounds was measured using a digital Shore A durometer, in accordance with ASTM D2240. Measurements were carried out at room temperature by applying a standardized force for a defined time through the specified indenter onto the flat surface of the specimens. The corresponding hardness values were recorded. For each rubber blend, five specimens were tested, and the reported values represent the average hardness.

### Tear test

The tear test was conducted to determine the maximum force required to propagate a tear along a constrained path (CP) in the rubber blend specimens. The test was performed in accordance with ASTM D624 using a universal testing machine at a constant crosshead speed of 50 ± 5 mm/min until complete specimen failure.

**Fig. 2:**
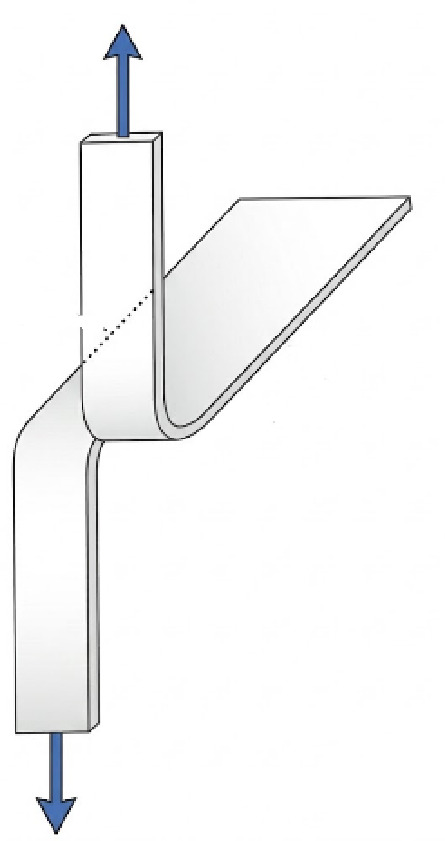
Schematic representation of the sample for measurement of the tear strength.

Tear strength was evaluated using the constrained path (CP) test specimens. An initial cut of 40 ± 1 mm was made between the two legs of each specimen using a sharp razor blade. The cut was made in a single stroke to ensure a sharp crack tip. The applied force required to propagate the tear was recorded, and the mean tear strength was calculated using the procedures specified in the standard. For each rubber compound, three specimens were tested. The peak tear force obtained from each test was used for analysis, and the average value was reported. A schematic representation of the CP test specimen is shown in Fig. 2.

### Abrasion test

Abrasion resistance refers to the ability of a material to withstand progressive material loss caused by mechanical actions such as rubbing, scraping, or erosion^40^. Abrasion resistance of the vulcanized rubber compounds was evaluated using a rotary drum abrasion tester in accordance with ISO 4649. Cylindrical specimens with dimensions of 16 mm in diameter and 6 mm in thickness were tested against a grade 60 abrasive sheet. During the test, the specimen was pressed against the rotating drum under a constant normal force of 10 N and a sliding speed of 0.32 m/s. The test was automatically terminated after the specimen had traversed a sliding distance of 40 m over the abrasive sheet. The abrasion behavior of the vulcanized rubber compounds was characterized in terms of relative volume loss (ΔV_rel_), which was calculated according to ISO 4649 using Eq. (4).

4 $$\:{\varDelta\:V}_{rel}=\:\left(\frac{{\varDelta\:m}_{t}-{\varDelta\:m}_{const}}{{\rho\:}_{t}\times\:{\varDelta\:m}_{r}}\right)$$

where Δm_t_ is the mass loss of the test specimen (mg), ρ_t_ is the density of the test specimen (mg/mm³), Δm_r_ is the mass loss of the reference rubber specimen (mg), and Δm_const_ is the abrasion constant specified for the reference rubber. The density of the vulcanized rubber compounds was determined using a densimeter according to ASTM D792.

### Thermogravimetric analyzer (TGA-DTG)

The thermal stability of the cured rubber compounds was evaluated using a simultaneous thermal analyzer (TGA-i-1000, THASS, Germany) capable of performing thermogravimetric (TGA) and derivative thermogravimetric (DTG) analyses. Before testing, the instrument was calibrated for temperature and heat flow using certified indium and zinc standards, while mass calibration was verified with built-in reference weights to ensure accuracy. Approximately 15 mg of each vulcanized NR/SBR sample was taken from the central region of the sheet to avoid surface oxidation effects and placed in an open platinum crucible^41,42^. All measurements were conducted in accordance with ASTM E1131. The samples were purged with high-purity nitrogen (99.99%) maintained at a controlled flow rate of 20 mL/min, ensuring a fully inert atmosphere and preventing oxidative or thermo-oxidative degradation. The mass loss was continuously recorded as a function of temperature. The total duration of the analysis was approximately 60 min.

The temperature program ranged from 25^◦^C and increased to 650^◦^C at a constant heating rate of 10^◦^C/min, a commonly accepted rate for polymeric and elastomeric materials to balance thermal lag and resolution^24^. The parameters extracted from the TGA and DTG curves were T_5_ (temperature at 5% mass loss), T_10_ (temperature at 10% mass loss), T_max_ (the maximum degradation rate from the DTG curve), and residue at 650 ^◦^C (%) recorded directly from the final mass plateau.

## Results and discussion

### Swelling test results

The swelling behavior and apparent crosslink density of the NR/SBR blends reinforced with carbon black CB N330 and N550 were evaluated by immersion in toluene following the procedure described in the Experimental section. In general, swelling measurements provide valuable insight into filler–rubber interactions and the effective network structure of vulcanized rubber compounds. Natural rubber (NR) and styrene–butadiene rubber (SBR) are nonpolar elastomers and therefore exhibit appreciable swelling in nonpolar solvents such as toluene^43,44^.

The results showed that the CB N330-filled blends generally exhibited lower swelling values than the corresponding CB N550-filled compounds. This behavior indicates stronger filler–rubber interactions and greater restriction of solvent penetration due to the higher surface area of N330 carbon black. In contrast, the CB N550-filled blends presented relatively higher swelling values, particularly at high SBR contents, suggesting weaker network restriction and increased chain mobility.

Apparent crosslink density is commonly estimated from equilibrium swelling measurements, where lower swelling values generally correspond to higher apparent crosslink density^45^. As shown in Table 3, the NR/SBR blends reinforced with CB N330 generally exhibited higher apparent crosslink density values than the corresponding N550-filled compounds. This behavior is attributed to stronger filler–rubber interactions and greater restriction of polymer chain mobility associated with the higher surface area of N330.

**Table 3:** Swelling ratio (ζ) and apparent crosslink density (ν_e_) of NR/SBR blends reinforced with carbon black N330 and N550.

SBR (phr)	CB N330 samples	Swelling ratio, ζ (%)	Crosslink density, $\:{{\upnu\:}}_{e}$ (×10⁻⁵ mol cm⁻³)	CB N550 samples	Swelling ratio, ζ (%)	Crosslink density, $\:{{\upnu\:}}_{e}$ (×10⁻⁵ mol cm⁻³)
**0**	A1	65.3 ± 0.42	8.89 ± 0.07	B1	69.6 ± 0.58	7.73 ± 0.09
**25**	A2	65.8 ± 0.36	8.82 ± 0.06	B2	65.2 ± 0.49	8.24 ± 0.08
**50**	A3	67.2 ± 0.51	8.45 ± 0.09	B3	67.0 ± 0.43	7.95 ± 0.07
**75**	A4	68.0 ± 0.47	8.18 ± 0.08	B4	70.1 ± 0.65	7.42 ± 0.11
**100**	A5	69.1 ± 0.55	7.96 ± 0.10	B5	80.8 ± 0.74	5.31 ± 0.13

It should be noted that, in filled rubber compounds, swelling behavior is influenced not only by the chemical crosslink density of the rubber network but also by filler–rubber and filler–filler interactions. Carbon black particles may act as additional physical crosslinking sites, restricting polymer chain mobility and solvent diffusion within the rubber matrix. Therefore, the apparent crosslink density obtained from swelling measurements should be regarded as an indicator of the overall network restriction rather than an absolute measure of the chemical crosslink density. This distinction is particularly important for carbon-black-filled rubber compounds, where filler-induced network effects may significantly influence solvent uptake behavior. The lower swelling and higher apparent crosslink density observed for the CB N330-filled blends suggest stronger filler–rubber interactions and a more constrained network structure compared with the corresponding CB N550-filled compounds.

The equilibrium swelling behavior was also found to be closely related to the mechanical performance and thermal stability of the investigated compounds. In general, compounds exhibiting lower swelling and higher apparent crosslink density tended to display higher elastic modulus values, indicating a more restricted polymer network. Crosslink density is also an important factor influencing the thermal stability of vulcanized rubber compounds, since greater energy is required to break the crosslinked network during thermal degradation^46^. The reduced swelling and increased apparent crosslink density observed for the CB N330-filled blends suggest the presence of a more restricted network structure, which may contribute to both enhanced mechanical performance and improved thermal stability. These relationships are further discussed in the subsequent sections.

Overall, these findings demonstrate that both carbon black type and NR/SBR blend composition significantly influence the effective network structure of the vulcanized compounds. The lower swelling and higher apparent crosslink density observed for the CB N330-filled blends suggest stronger filler–rubber interactions and a more restricted network structure compared with the corresponding CB N550-filled compounds. Similar observations have been reported previously, where fillers with smaller particle sizes were found to enhance filler–rubber interactions and improve the physico-mechanical properties of rubber composites^47–49^. These effects are reflected in the mechanical and thermal characteristics discussed in the following sections.

### Mechanical properties

Table 4 summarizes the mechanical and abrasion properties of the investigated NR/SBR blends reinforced with carbon black N330 and N550, highlighting the combined effects of blend composition and filler type on the performance of the vulcanized compounds. In general, increasing the SBR content reduced the tensile strength, elongation at break, toughness, and tear strength of the blends. In contrast, the tensile modulus, hardness, and abrasion resistance exhibited composition-dependent behavior. At all blend compositions, the N330-filled compounds generally exhibited superior mechanical performance and abrasion resistance compared with the corresponding N550-filled blends, reflecting the higher reinforcing efficiency of N330 carbon black.

**Table 4:** Summary of the mechanical and abrasion properties of NR/SBR blends reinforced with carbon black N330 and N550 (mean ± standard deviation).

SBR (phr)	Tensile strength (MPa)		Elongation at break (%)		Tensile modulus (MPa)		Toughness (MJ/m³)		Hardness (Shore A)		Tear strength (*N* mm⁻¹)		Relative volume loss (mm³)	
	N330	N550	N330	N550	N330	N550	N330	N550	N330	N550	N330	N550	N330	N550
0	23.65 ± 0.60	19.71 ± 2.11	745.01 ± 5.00	691.21 ± 6.06	2.31 ± 0.18	2.15 ± 0.15	76.70 ± 1.19	59.88 ± 2.76	68.85 ± 2.17	53.58 ± 5.88	18.50 ± 1.16	12.75 ± 1.30	46.49 ± 1.04	49.62 ± 1.39
25	20.58 ± 1.51	17.51 ± 1.50	574.84 ± 4.33	506.82 ± 4.17	3.06 ± 0.16	2.90 ± 0.19	54.45 ± 3.89	37.13 ± 3.19	72.50 ± 2.62	59.35 ± 5.05	17.50 ± 1.45	12.01 ± 1.20	44.83 ± 1.50	50.11 ± 0.34
50	16.10 ± 1.08	13.94 ± 0.75	474.80 ± 3.63	461.48 ± 5.15	3.01 ± 0.18	2.82 ± 0.16	34.70 ± 2.35	30.07 ± 1.29	70.65 ± 2.57	66.15 ± 4.17	14.62 ± 1.50	6.13 ± 0.63	51.21 ± 1.16	55.15 ± 1.25
75	15.52 ± 1.86	13.00 ± 1.08	471.39 ± 2.17	466.06 ± 1.81	2.87 ± 0.18	2.56 ± 0.16	33.05 ± 3.65	27.77 ± 1.15	68.50 ± 3.52	67.77 ± 3.16	9.10 ± 1.00	4.80 ± 1.00	52.15 ± 2.34	55.80 ± 1.04
100	13.11 ± 1.35	11.50 ± 0.28	531.04 ± 3.93	577.38 ± 6.66	2.03 ± 0.13	1.69 ± 0.12	30.53 ± 3.67	29.83 ± 0.80	66.19 ± 3.47	62.54 ± 2.53	11.50 ± 0.71	10.88 ± 1.20	54.23 ± 1.48	58.49 ± 2.62

#### Tensile strength

**Fig. 3:**
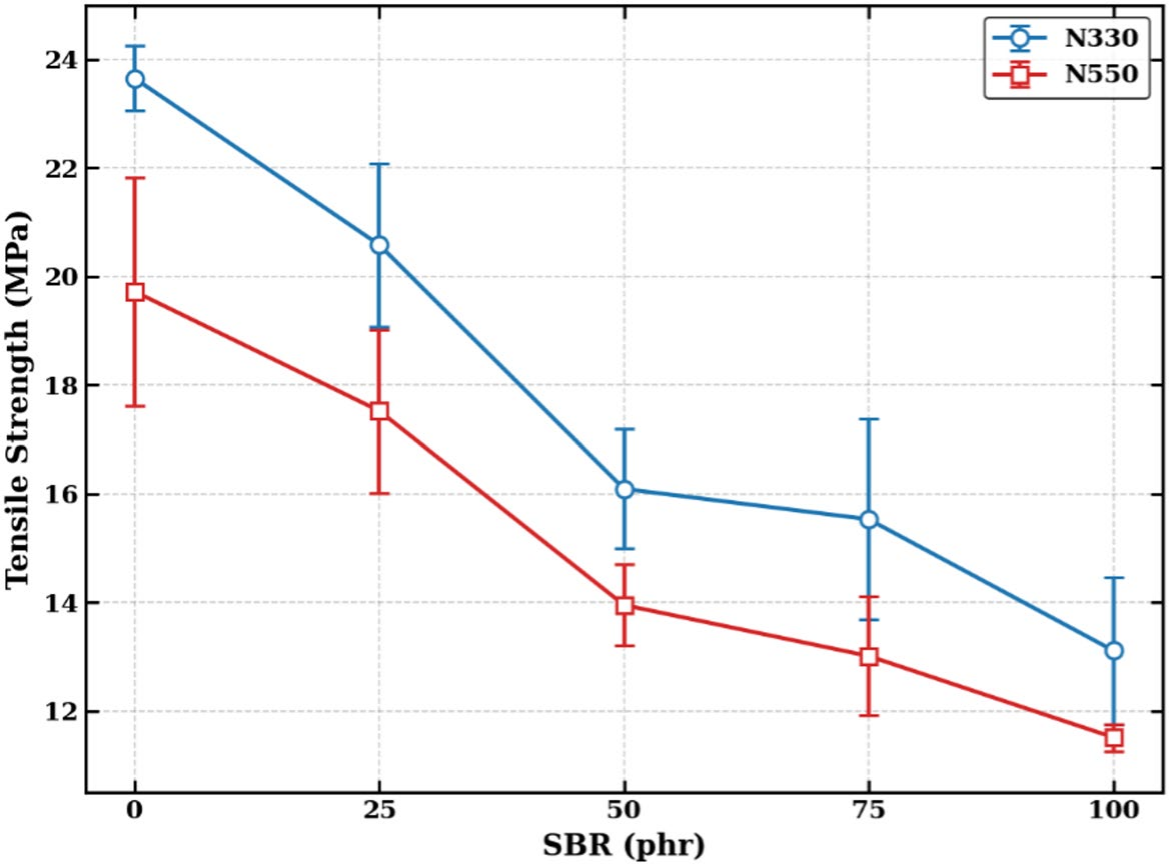
Tensile strength of NR/SBR blends as a function of SBR content (average values ± standard deviation).

The results showed significant differences between NR/SBR rubber blends reinforced with CB N330 (A-series) and N550 (B-series). Figure 3 illustrates the relationship between the tensile strength of the rubber blends and the SBR content for both compounds filled with CB N330 and N550. All blends reinforced with CB N330 (A1–A5) consistently showed higher tensile strength values than those filled with CB N550 (B1–B5).

A systematic decrease in tensile strength is observed with an increase in the SBR content for both filler systems. This behavior reflects the progressive replacement of natural rubber, which exhibits strain-induced crystallization, by styrene–butadiene rubber, an essentially amorphous elastomer with limited crystallization capability under tensile deformation. In addition to the variation in NR/SBR blend ratio and filler properties, tensile strength is influenced by the density of crosslinks within the rubber network^50,51^. For the CB N330-filled rubber blends, compound A1 exhibited the highest tensile strength (23.65 MPa). Compared to A1, the tensile strength of compounds A2-A5 decreased by 12.98%, 31.92%, 34.38% and 44.58%, respectively, as the SBR content increased from 25 to 100 phr. Similarly, for the N550-filled blends, compound B1 showed the highest tensile strength (19.71) MPa. Compared to B1, the tensile strength of compounds B2-B5 decreased by 11.16%, 29.27%, 34.05% and 41.65%, respectively, with increasing SBR content.

The higher tensile strength of the CB N330-filled blends is consistent with the swelling results, which revealed stronger filler–rubber interactions and higher apparent crosslink density. The larger interfacial area and more developed structure of CB N330 promote bound rubber formation and stronger adhesion between the filler and rubber matrix, improving stress transfer and enhancing the integrity of the network^52–55^. Polymer chains strongly attached to carbon black particles may act as additional physical crosslinks, contributing to the improved tensile properties^56–58^.

The reduction in tensile strength with decreasing NR while increasing SBR content further highlights the dominant role of natural rubber in sustaining high tensile properties through strain-induced crystallization, which provides additional reinforcement during deformation and delays fracture^59^. As the NR fraction decreases, this reinforcing mechanism becomes less effective, resulting in lower tensile strength despite the presence of reinforcing fillers. Overall, the tensile strength of the investigated blends is governed by the combined effects of the NR/SBR blend ratio, strain-induced crystallization of natural rubber, and the reinforcing mechanism associated with carbon black.

#### Tensile modulus

**Fig. 4:**
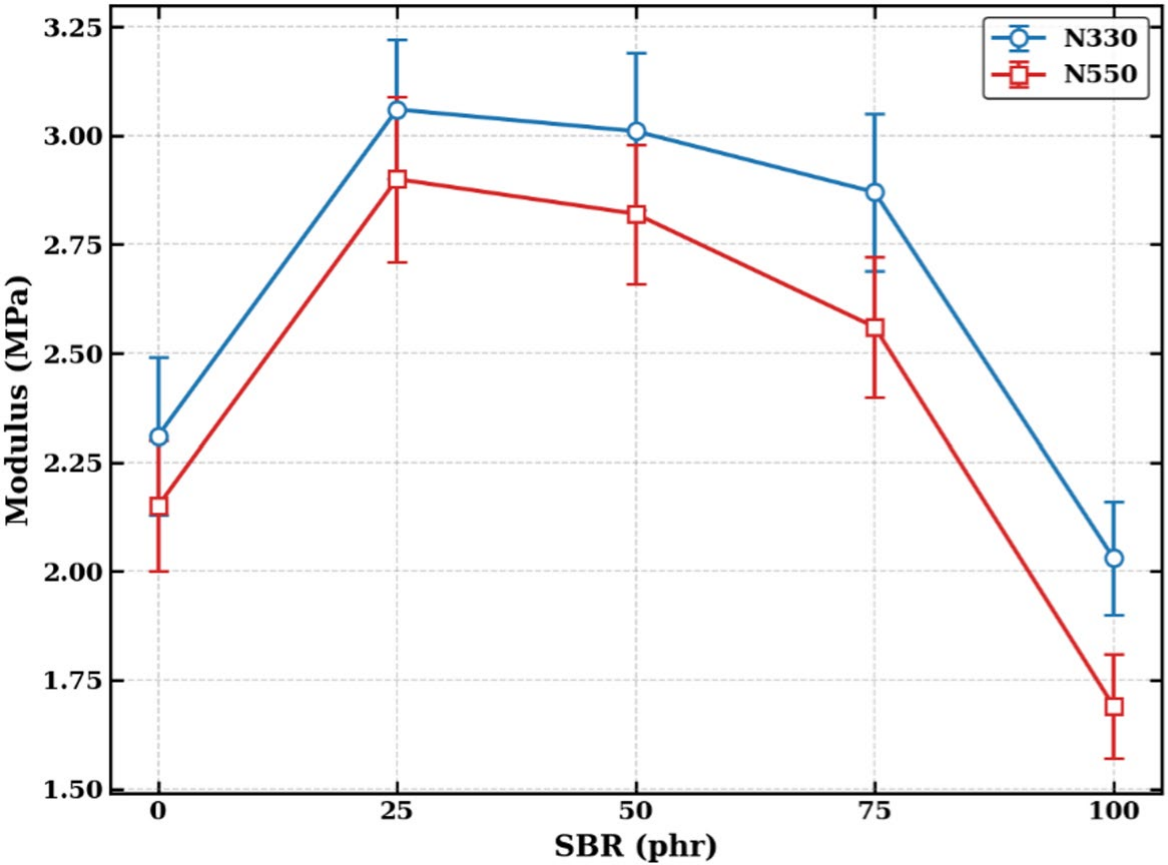
Relationship between tensile modulus of NR/SBR blends as a function of SBR (average values ± standard deviation).

Figure 4 illustrates the relationship between the tensile modulus of NR/SBR blends and SBR content for compounds reinforced with CB N330 and N550. All blends reinforced with CB N330 (A1–A5) consistently showed higher tensile modulus values than those filled with CB N550 (B1–B5), highlighting the stronger reinforcing effect associated with N330 carbon black under the present experimental conditions.

For the CB N330-filled blends, compound A2 exhibited the highest tensile modulus (3.06 MPa), while both pure NR (A1) and pure SBR (A5) showed comparatively lower modulus values than the intermediate blend compositions. Similarly, compound B2 exhibited the highest tensile modulus among the CB N550-filled blends, whereas compounds B1 and B5 showed comparatively lower modulus values. These results are consistent with the findings reported by Roslee et al.^60^, which also showed that intermediate NR/SBR blend ratios displayed higher tensile modulus values than the corresponding pure rubber compounds. The higher modulus observed for the intermediate blend compositions suggests that the combination of NR and SBR provides a favorable balance between elasticity and network rigidity, resulting in greater resistance to deformation than the corresponding pure rubber compounds.

The tensile modulus of the investigated blends is influenced not only by the particle size and surface area of carbon black but also by its structural characteristics and reinforcing capability^61–64^. These factors influence the reinforcing behavior of the vulcanizates and contribute to their stiffness and resistance to deformation. Crosslink density also strongly influences the tensile modulus of vulcanized rubber. An increase in the number of crosslinks restricts the mobility of polymer chains and reduces their ability to undergo deformation, thereby increasing the stiffness and tensile modulus of the rubber network^65^.

The swelling analysis further supports these results, revealing that the CB N330-filled blends demonstrated higher apparent crosslink density and a more restricted network structure than the corresponding N550-filled compounds. Thus, the higher apparent crosslink density and restricted network structure of the CB N330-filled blends contributed to their greater resistance to deformation and higher tensile modulus values compared with the corresponding N550-filled compounds^65,66^.

#### Elongation at break

**Fig. 5:**
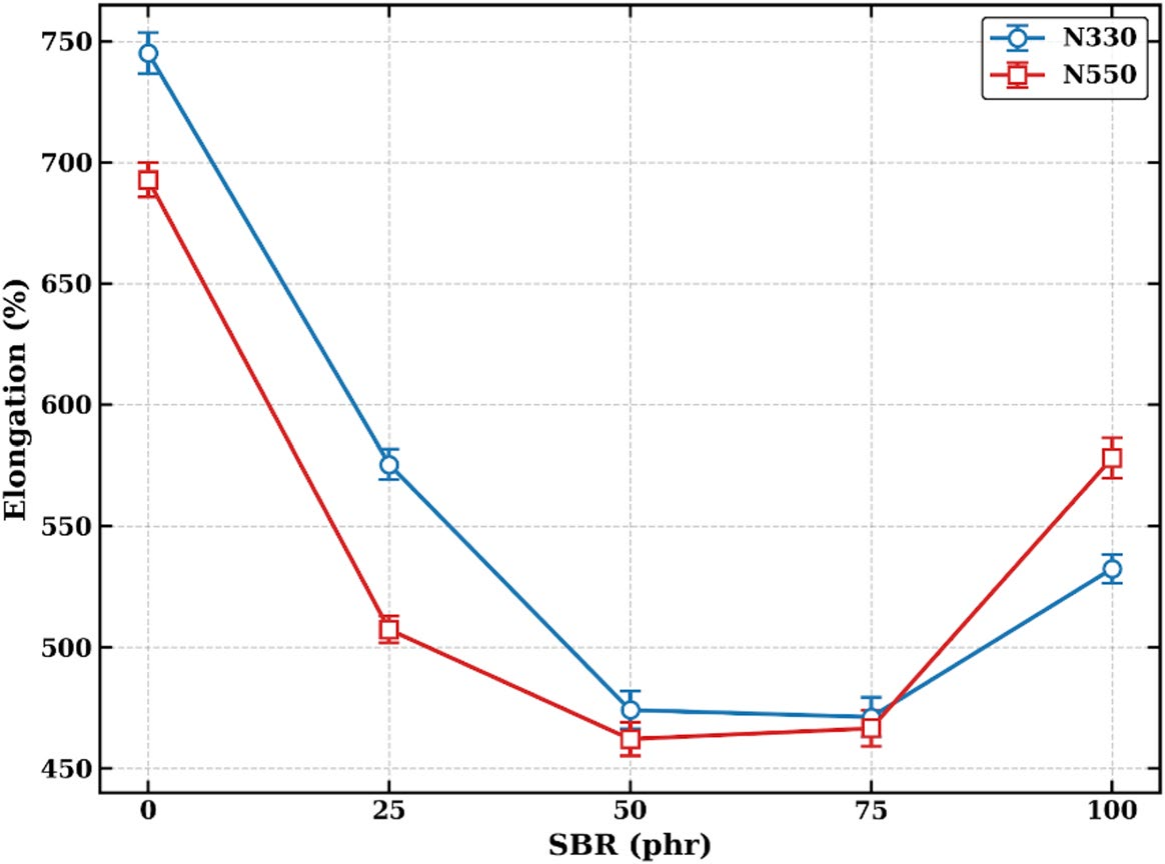
Elongation at break of NR/SBR blends as a function of SBR (average values ± standard deviation).

The results of elongation at break presented in Fig. 5 show a clear dependence on carbon black grade and NR/SBR composition. The highest value was observed for blend A1 (745.01%). This value decreased with increasing SBR content in blends A2-A5. This behavior is characteristic of natural rubber because the degree of elasticity, the density of cross-linking formed, and molecular chain length all directly affect elongation at break^67,68^. The higher elongation of NR-rich blends is associated with the ability of natural rubber chains to undergo strain-induced crystallization, which delays crack propagation and allows larger deformation before failure.

For the intermediate NR/SBR blends reinforced with CB N330 (A2, A3, and A4), the elongation at break was slightly higher than that of the corresponding CB N550-filled blends (B2, B3, and B4). For A5 and B5, a partial increase in elongation at break was observed for both CB N330 and N550 NR/SBR compounds, with the CB N550-filled blend presenting a slightly higher elongation. The partial increase observed for the pure SBR compounds may be associated with the greater chain flexibility of SBR, which allows larger deformation before fracture despite its limited strain-induced crystallization capability. In addition, differences in carbon black grade may influence polymer chain mobility and deformation behavior, thereby affecting the elongation at break of the investigated compounds^58,69,70^.

#### Toughness

**Fig. 6:**
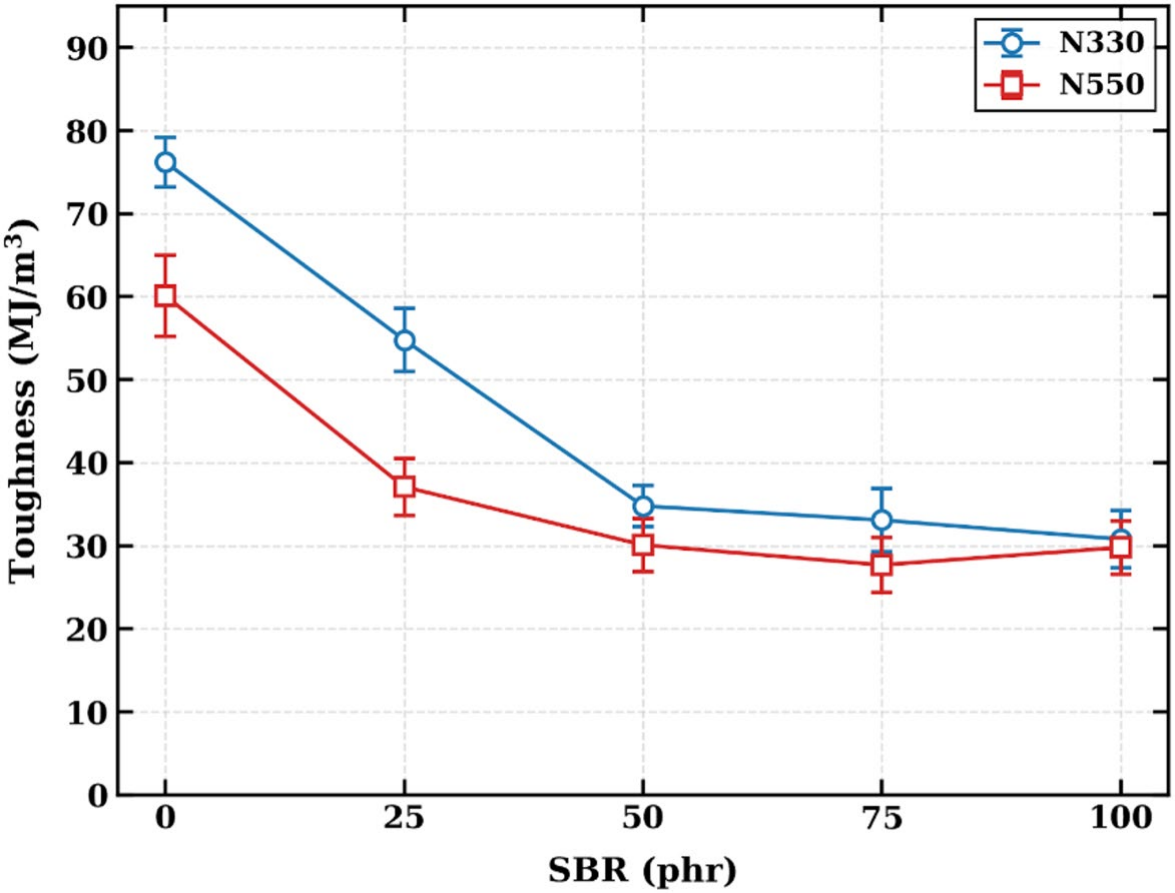
Toughness of NR/SBR blends reinforced with carbon black N330 and N550 as a function of SBR content (average values ± standard deviation).

Toughness, represented by the area under the stress–strain curve, corresponds to the energy absorbed per unit volume before fracture and is governed by the combined contributions of tensile strength and elongation at break^71–73^. The CB N330-filled blends (A1–A5) consistently illustrated higher toughness values than the corresponding CB N550-filled compounds (B1–B5), indicating superior energy absorption capability, as shown in Fig. 6. For the CB N330-filled blends, compound A1 showed the highest toughness, and toughness gradually decreased with increasing SBR content. This reduction in toughness reflects the simultaneous decreases in tensile strength and elongation at break observed for the investigated blends, as reductions in these parameters directly decrease the energy absorbed before fracture.

In rubber compounds, toughness depends on achieving an appropriate balance between reinforcement and deformability^74,75^. High stiffness and excellent toughness are often competing requirements in elastomeric materials^76–78^. Conventional reinforcement strategies based on increased filler loading or higher crosslink density improve modulus and stress transfer but may restrict polymer chain mobility and reduce the ability of the material to absorb energy before fracture, leading to lower toughness^52,79,80^.

Hence, the overall toughness of elastomeric materials is governed by the relationship between network reinforcement and the ability of polymer chains to undergo large deformations. Effective filler–rubber interactions improve stress transfer between the rubber matrix and the reinforcing filler, promoting a more uniform stress distribution and delaying crack initiation and propagation. Therefore, greater energy is required for fracture, leading to improved toughness^81^.

In addition, the progressive replacement of natural rubber with SBR reduces the contribution of strain-induced crystallization, thereby diminishing the resistance of the blends to crack growth during deformation^82,83^. As a result, the ability of the material to absorb energy before fracture decreases, leading to lower toughness. Therefore, compound A5 exhibited the lowest toughness among the CB N330-filled blends. This behavior further emphasizes the important contribution of strain-induced crystallization in maintaining the energy absorption capability of NR-rich compounds.

Similarly, compound B1 revealed the highest toughness among the CB N550-filled blends, followed by a progressive decrease as the SBR content increased. The comparatively lower toughness of the N550-filled blends reflects the lower reinforcement imparted by N550 carbon black^84^. For the 100% SBR formulation (B5), a slight increase in toughness was observed compared with B4. This behavior may be attributed to the relatively higher elongation at break of the SBR matrix, which partially compensates for the reduction in tensile strength and contributes to greater energy absorption during deformation. Overall, the CB N330-filled NR/SBR blends demonstrated a greater ability to absorb deformation energy before fracture than the corresponding N550-filled blends^85,86^.

#### Tear strength

**Fig. 7:**
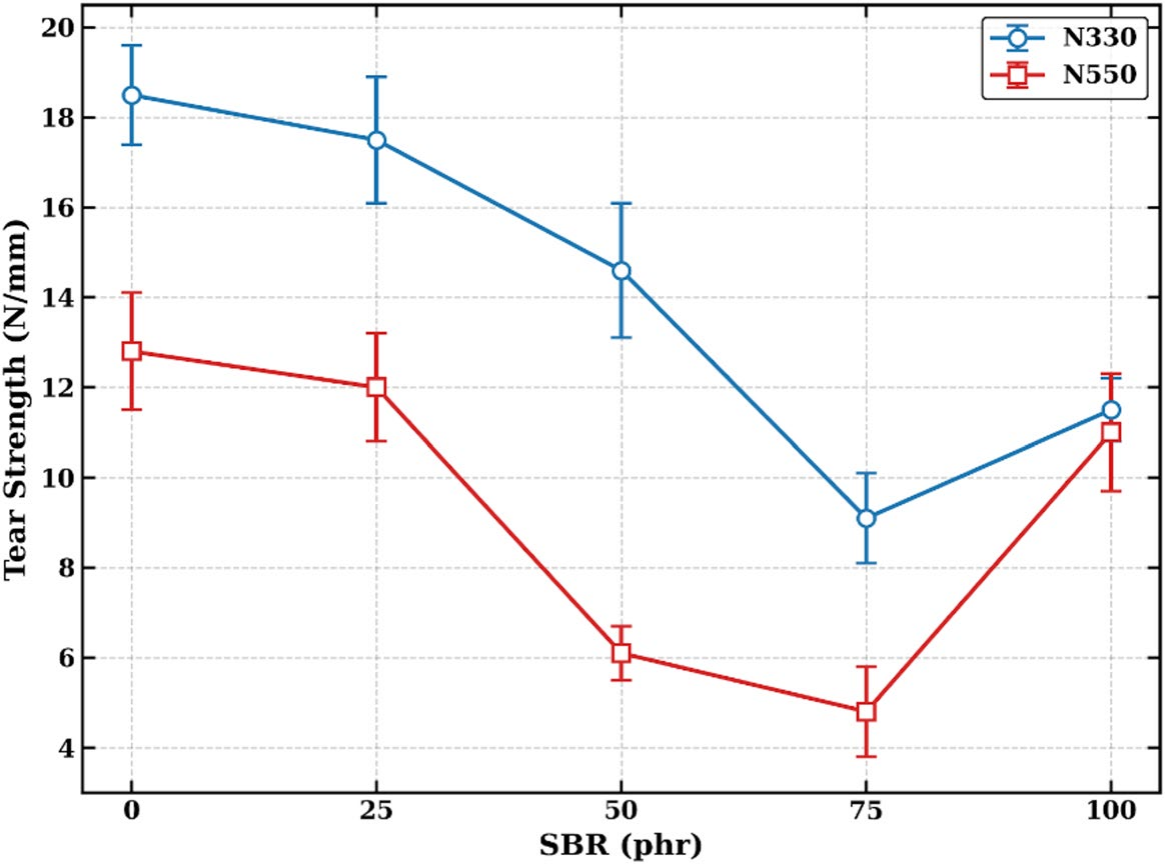
Tear strength of NR/SBR blends reinforced with carbon black N330 and N550 (average values ± standard deviation).

Tear strength is an important mechanical property that reflects the ability of a rubber compound to resist crack initiation and propagation under applied stress. High tear strength contributes to longer service life in elastomeric components by improving the ability of the material to resist the propagation of cuts or flaws under tensile loading^87,88^. Lower tear strength facilitates crack propagation and premature failure, thereby reducing the service life and reliability of elastomeric components. Figure 7 shows the tear strength of different NR/SBR blends reinforced with CB N330 and N550 using peak-only analysis in accordance with ASTM D624.

The tear strength of NR/SBR blends filled with CB N330 and N550 strongly depends on both blend composition and carbon black grade. The NR/SBR formulations reinforced with CB N330 consistently exhibited higher tear strength than the corresponding blends filled with CB N550, indicating the greater reinforcement imparted by CB N330. The variation in tear strength is strongly influenced by the relative proportions of NR and SBR, as each elastomer contributes differently to deformation behavior, crack-growth resistance, and reinforcement efficiency^89,90^.

The smaller particle size and higher specific surface area of CB N330 promote stronger filler–rubber interactions and improved interfacial adhesion, which enhance stress transfer between the rubber matrix and the reinforcing filler. Consequently, stress concentration around crack tips is reduced, delaying crack initiation and propagation and requiring greater energy for tear growth^91,92^.

Among all formulations, compound A1 displayed the highest tear strength (17.5 N/mm), followed by a progressive decrease with increasing SBR content up to 75 phr. The reduction in tear strength with increasing SBR content may be attributed to the lower NR content and the corresponding reduction in strain-induced crystallization during deformation. Strain-induced crystallization of NR locally reinforces the material around the crack tip, thereby delaying crack propagation and enhancing tear resistance^93–95^. As a result, NR-rich blends generally exhibited higher tear strength than SBR-rich blends^95,96^.

For compound A5, a partial increase of 26.24% in tear strength was observed compared with A4. On the other hand, the lowest tear strength (4.13 N/mm) was observed for compound B4, followed by a substantial increase in compound B5 as the SBR content increased from 75 to 100 phr. This behavior may be associated with the deformation characteristics of the SBR matrix and its relatively high elongation at break, which can contribute to greater energy dissipation during tearing and partially compensate for the reduction in tensile strength^97–99^. This behavior is consistent with the elongation-at-break results, where the pure SBR compounds exhibited a partial recovery in ductility. Nevertheless, the tear strength values of the CB N550-filled blends remained lower than those of the corresponding CB N330-filled compounds, reflecting the lower reinforcement imparted by N550 carbon black. Tear strength is also influenced by tensile strength, elongation at break, hardness, filler–rubber interactions, and network structure^27,100,101^. The greater network restriction associated with higher apparent crosslink density may contribute to improved tear resistance by limiting chain mobility and increasing resistance to crack growth^102^.

Overall, the observed trends indicate that tear resistance is governed by the combined effects of filler–rubber interactions, apparent crosslink density, strain-induced crystallization, and resistance to crack initiation and propagation. The stronger reinforcing characteristics of N330 contributed to superior tear strength compared with the corresponding N550-filled compounds through enhanced filler–rubber interactions, improved interfacial adhesion, more efficient stress transfer, and increased resistance to crack growth^103^.

#### Hardness

**Fig. 8:**
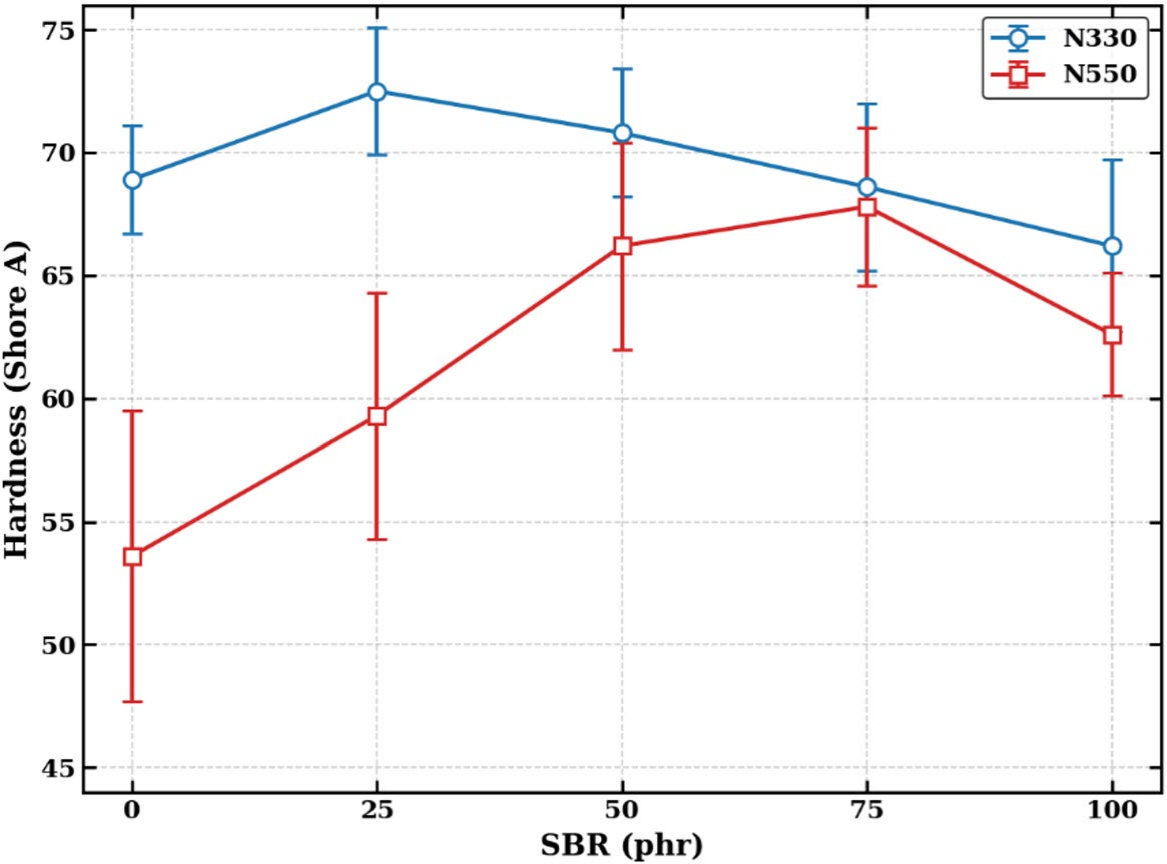
Hardness of the NR/SBR blends (average values ± standard deviation).

Hardness is a measure of the resistance of rubber to localized reversible deformation under the action of a rigid indenter and is commonly used as a quality control factor. The hardness of the rubber compounds was measured using a Shore A durometer in accordance with ASTM D2240. The obtained results are presented in Fig. 8. The hardness values of the investigated compounds ranged from 53 to 72 Shore A.

The results show that the hardness of the CB N330-filled blends was constantly higher than that of the corresponding CB N550-filled compounds, as illustrated in Fig. 8. The highest hardness observed for compound A2 suggests that intermediate NR/SBR compositions provide a favorable balance between elasticity and network rigidity, resulting in increased resistance to localized deformation. Similar behavior has been reported previously for NR/SBR blends, where intermediate blend ratios exhibited higher hardness than the corresponding pure rubber compounds^104^. This trend is also consistent with the tensile modulus results, where intermediate blend compositions exhibited greater stiffness than the corresponding pure NR and pure SBR compounds.

The higher hardness of the CB N330-filled blends is consistent with the reinforcement mechanism established in the swelling study, which indicated stronger filler–rubber interactions and higher apparent crosslink density for the CB N330-filled compounds. Hardness is strongly influenced by the mobility of polymer chains and the stiffness of the rubber network. Stronger filler–rubber interactions increased apparent crosslink density, restricted segmental motion, and increased resistance to localized deformation, thereby producing higher hardness values^105,106^.

Furthermore, polymer chains strongly attached to the filler surface in the form of bound rubber may act as additional physical constraints, further restricting chain mobility and contributing to increased hardness. The hardness behavior of the investigated compounds reflects the combined effects of apparent crosslink density, bound rubber formation, and the stiffness of the rubber network.

#### Abrasion resistance

**Fig. 9:**
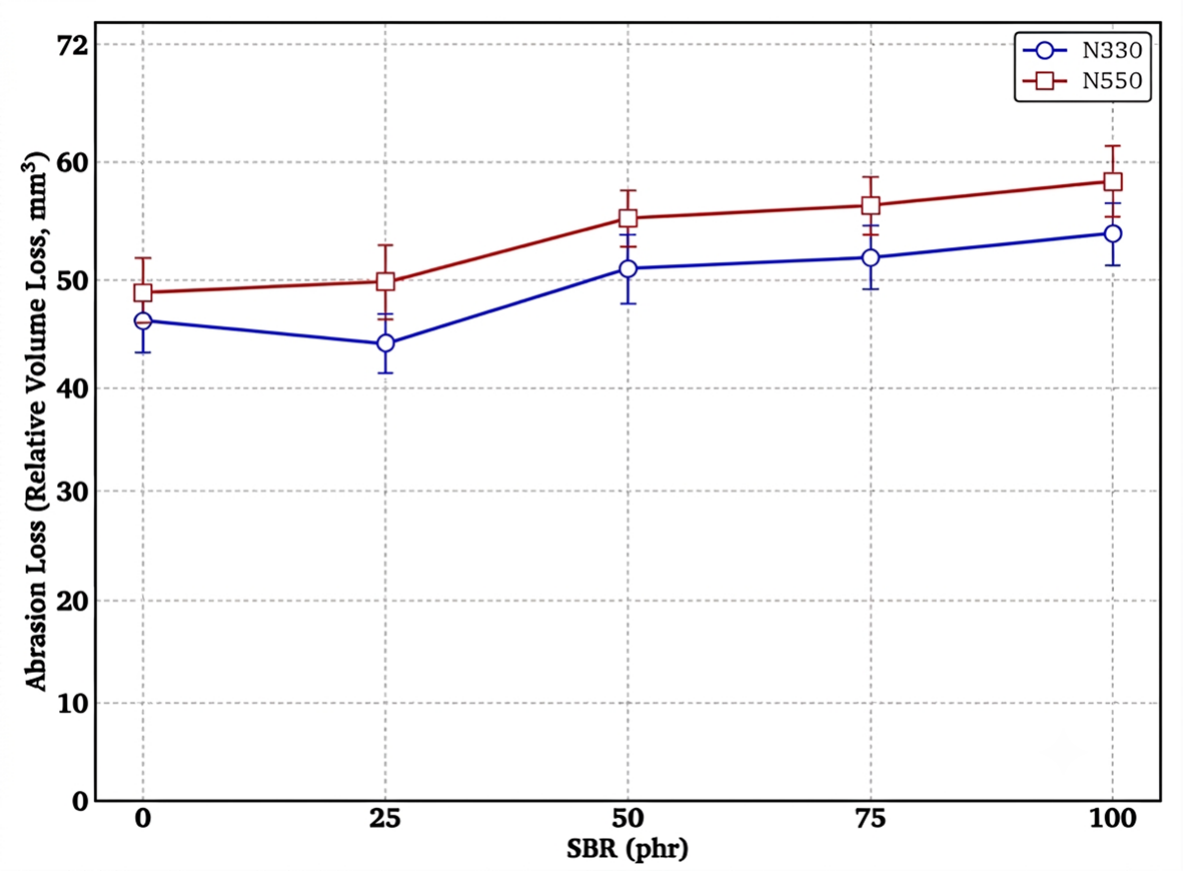
Relative volume loss of NR/SBR blends reinforced with carbon black N330 and N550 as a function of SBR content (average values ± standard deviation).

To evaluate the abrasion resistance of the NR/SBR compounds, abrasion testing was performed in accordance with ISO 4649 using a DIN abrasion resistance tester. Abrasion behavior was evaluated by measuring the relative volume loss. Figure 9 presents the relative volume loss of the NR/SBR blends reinforced with carbon black N330 and N550 as a function of SBR content.

The results show that the CB N330-filled blends consistently exhibited lower relative volume loss than the corresponding CB N550-filled blends at all blend compositions, demonstrating higher abrasion resistance. Among the investigated compounds, the NR/SBR (75/25) blend reinforced with CB N330 showed the lowest relative volume loss (44.83 ± 1.50 mm³), indicating the highest abrasion resistance. Beyond 25 phr SBR, the relative volume loss generally increased for both filler systems, indicating a reduction in abrasion resistance. This behavior is consistent with the decrease in tensile strength and tear strength accompanying the reduction in NR content and the associated loss of strain-induced crystallization. Furthermore, the higher abrasion resistance of the CB N330-filled blends is attributed to the superior reinforcing characteristics of CB N330, including its smaller particle size, higher specific surface area, and stronger filler–rubber interactions. Similar improvements in abrasion resistance for CB N330-filled rubber compounds have been reported previously^107^.

The abrasion behavior of rubber compounds is governed by several interacting factors, including tensile strength, tear strength, hardness, filler–rubber interactions, and network structure^108^. Higher tensile and tear strength generally improve resistance to crack initiation and propagation during repeated surface deformation and frictional contact, whereas increased hardness enhances resistance to material removal and surface wear. Therefore, the superior mechanical performance observed for the CB N330-filled blends, particularly in terms of tensile strength, tear strength, and hardness, is consistent with the superior abrasion resistance measured experimentally.

In addition, increased apparent crosslink density enhances network integrity, restricts polymer chain mobility, and strengthens intermolecular interactions, thereby contributing to improved dynamic properties and abrasion resistance^25^. Materials with low tear resistance generally exhibit poor abrasion resistance because surface damage can propagate more readily under repeated mechanical loading^108^. However, although increased hardness and apparent crosslink density generally improve abrasion resistance, excessive network restriction may reduce toughness and limit the material’s ability to dissipate mechanical stresses through elastic deformation. This may promote localized stress concentrations and result in slightly higher abrasion loss, consistent with previous studies^109,110^.

Moreover, the finer particle size and higher specific surface area of CB N330 promote more uniform filler dispersion, increase the effective filler–polymer interfacial area, and improve stress transfer throughout the rubber matrix. Improved filler dispersion reduces the formation of stress concentration sites associated with filler agglomerates, thereby promoting a more homogeneous stress distribution during repeated abrasive loading. Consequently, crack initiation is delayed, and crack propagation is suppressed, reducing material removal from the rubber surface and resulting in lower relative volume loss^111,112^.

Overall, the abrasion resistance of the investigated compounds was governed by the combined effects of filler–rubber interactions, filler dispersion, apparent crosslink density, strain-induced crystallization, and resistance to crack initiation and propagation. Accordingly, the superior tensile strength, tear strength, hardness, and apparent crosslink density of the CB N330-filled blends are consistent with their lower relative volume loss measured during abrasion testing, confirming the superior reinforcing efficiency of CB N330 compared with CB N550.

### Thermogravimetric analysis (TGA)

**Fig. 10:**
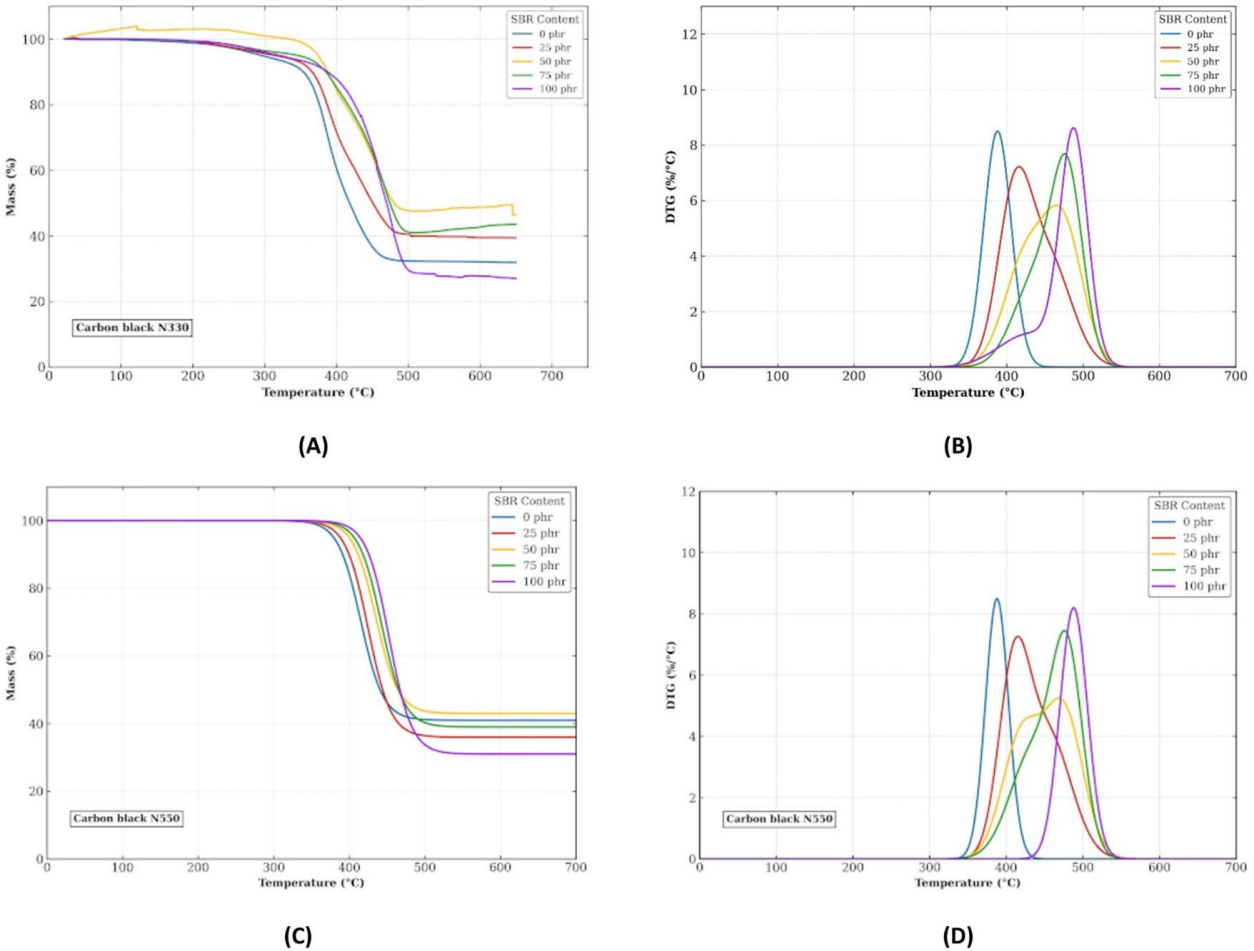
Thermogravimetric analysis (TGA) and derivative thermogravimetric (DTG) curves of NR/SBR blends reinforced with different carbon black grades: (**A**) TGA and (**B**) DTG curves for Series N330; (**C**) TGA and (**D**) DTG curves for Series N550.

Thermal stability studies, which analyze how elastomers respond to temperature changes, play a key role in understanding their degradation patterns and lifespan^113^. Thermogravimetric analysis (TGA) is widely used to measure the thermal stability and study the degradation behavior of polymeric materials due to the simplicity of the mass-loss method^114,115^. The derivative thermogravimetric (DTG) curves of the prepared NR/ SBR blends for series A (N330) and series B (N550) are presented in Fig. 10 (A, B, C, and D). Polymers are often exposed to high temperatures during processing and use. Thus, thermal stability is one of the most important properties for a wide range of applications^116^.

The TGA curves indicate that the thermal degradation of the prepared NR/SBR blends occurs in a single stage, indicating a homogeneous degradation mechanism. No significant mass loss was observed below 100 °C, indicating a negligible moisture content in the investigated compounds^117^. A gradual mass loss up to approximately 290 °C may be attributed to the evaporation of semi-volatile constituents, including extender oils, softeners, antioxidants, and residual vulcanization additives. The major mass loss observed between approximately 300 and 500 °C was attributed to the thermal decomposition of the NR/SBR rubber matrix^118,119^. In this temperature range, polymer chain scission, depolymerization, and volatilization of low-molecular-weight degradation products occur, resulting in the principal degradation stage of the vulcanized compounds^120^. No significant mass loss was observed beyond approximately 500 °C, where the remaining residue mainly consists of carbon black, inorganic constituents, and other non-rubber components that do not volatilize under a nitrogen atmosphere, leaving behind solid residue content^18,121^.

From the TGA analysis, it was observed that the T₁₀ values increased with increasing SBR content for both filler systems, indicating improved thermal stability. In contrast, the variation in T₅ depended on filler type and blend composition. For the N330-filled blends, T₅ increased from 295.53 °C (0 phr SBR) to 321.36 °C (100 phr SBR), while T₁₀ increased from 353.82 °C to 387.67 °C. Similarly, for the N550-filled blends, T₅ ranged from 317.01 °C to 309.50 °C, and T₁₀ increased from 356.62 °C to 384.85 °C with increasing SBR content.

**Fig. 11:**
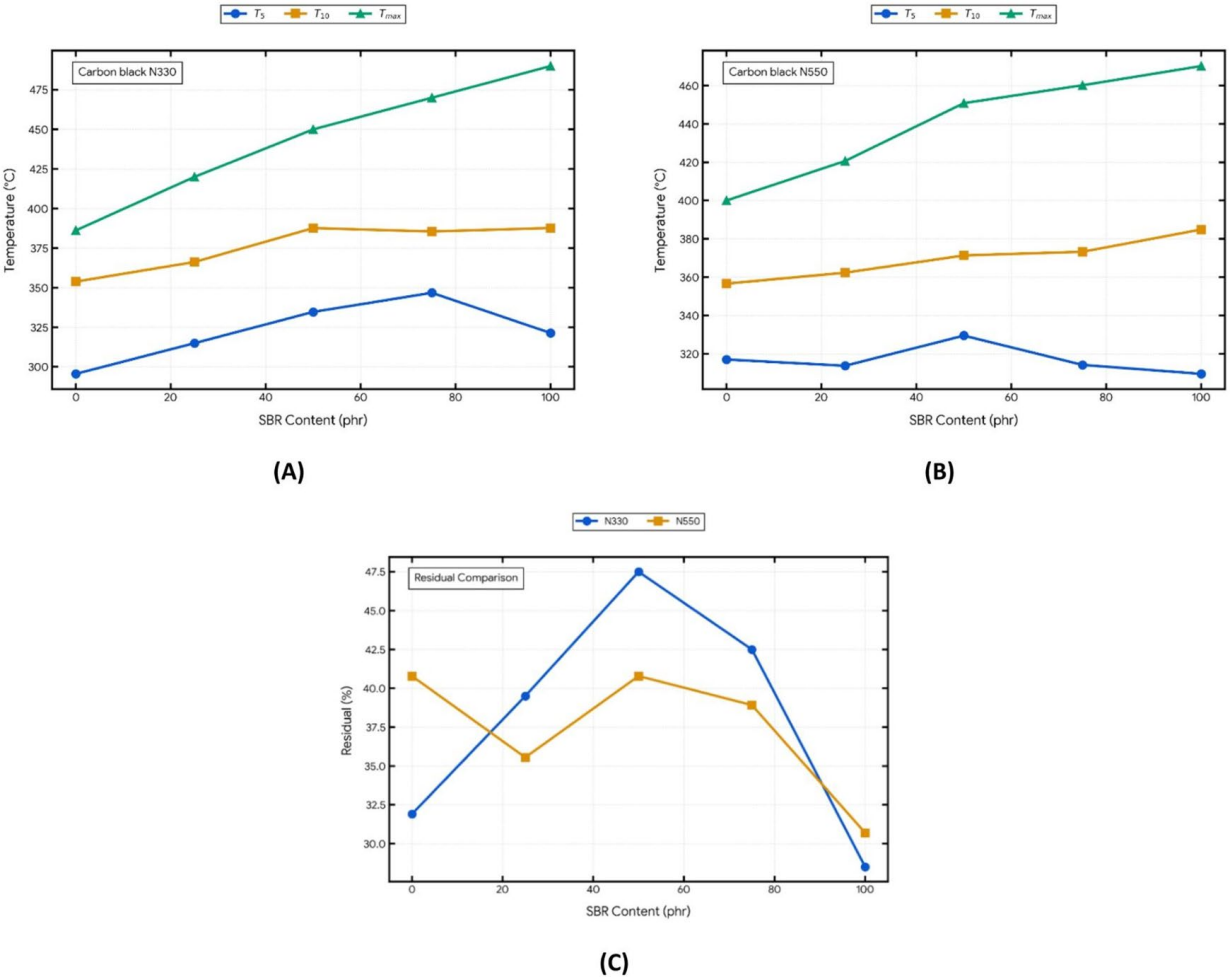
Thermal stability parameters (T₅, T₁₀, and Tₘₐₓ) and residual mass of NR/SBR blends reinforced with (**A**) carbon black N330, (**B**) carbon black N550, and (**C**) a comparison of their residual mass.

The DTG curves confirm the single-step degradation behavior, showing a single, well-defined peak (Tₘₐₓ) for all compositions within the range of approximately 380–490 °C. The DTG peaks correspond to the maximum rate of mass loss^122^. This indicates that both the NR and SBR phases degrade simultaneously rather than independently. It is also observed that Tₘₐₓ shifts progressively toward higher temperatures as SBR content increases, indicating enhanced thermal stability of the blends. This trend is supported by the increase in T₁₀ values with increasing SBR content, as presented in Fig. 11 (A, B, and C).

The analysis of the thermal stability of NR/SBR blends filled with carbon black N330 (Series A) and N550 (Series B) shows a clear effect of filler type on degradation behavior. For both systems, increasing the SBR content leads to a progressive shift of T₁₀ and Tₘₐₓ toward higher temperatures, confirming enhanced thermal stability of the blends. However, notable differences are observed between the two fillers. The NR/SBR blends reinforced with CB N330 exhibit higher residual mass, particularly at intermediate SBR contents, indicating greater char formation. This behavior is attributed to the higher specific surface area of CB N330, and the reinforcement mechanism established in the swelling study, which indicated stronger filler–rubber interactions, higher apparent crosslink density, and greater network restriction. On the other hand, the NR/SBR blends filled with CB N550 display slightly higher initial decomposition temperatures (T₅) and a more gradual increase in Tₘₐₓ, suggesting a slightly more gradual degradation behavior.

Although heat buildup was not directly evaluated in the present study, previous studies have reported that carbon black surface area, filler structure, and network characteristics significantly influence energy dissipation and heat generation in rubber compounds^123–125^. Higher carbon black surface area and filler structure have been associated with stronger filler–rubber interactions, which may contribute to increased hysteresis and heat buildup through greater energy dissipation during cyclic deformation. Heat generation in elastomers is also influenced by network structure and crosslink density. In general, increasing crosslink density restricts polymer chain mobility and may reduce hysteretic losses during repeated deformation. Excessively high crosslink densities, however, may lead to increased brittleness, indicating that an optimum network structure is required for balanced mechanical performance and resistance to heat buildup^126–129^. Nevertheless, the overall heat buildup behavior depends on the interplay between filler characteristics and network structure^126,128^. These competing effects suggest that heat generation behavior is governed by a complex balance between filler-induced energy dissipation and network restriction. Consequently, heat buildup behavior cannot be inferred solely from thermal stability measurements, and no direct conclusions regarding hysteresis or heat generation can be drawn from the present TGA results.

Although TGA evaluates thermal degradation under non-service conditions, the obtained thermal stability parameters provide useful information regarding the resistance of rubber compounds to thermally induced degradation at elevated temperatures^118^. It should be noted that the thermal stability parameters and residual mass obtained from TGA primarily reflect the resistance of the compounds to thermal decomposition and char formation under the applied test conditions.

In the present study, the CB N330-filled blends exhibited higher residual mass than the corresponding CB N550-filled compounds. Furthermore, the higher residual mass of the CB N330-filled blends indicates increased char formation and greater resistance to thermal decomposition under the conditions employed in the TGA analysis. These characteristics suggest enhanced thermal robustness at elevated temperatures, which is consistent with the higher apparent crosslink density obtained from the swelling experiments.

TGA and DTG analyses confirmed that increasing the SBR content improved the thermal stability of the investigated blends, which may be attributed to the higher thermal stability of SBR compared with pure NR^46,130^. The thermal stability of the vulcanized compounds was influenced by polymer composition and filler characteristics. In addition, the higher residual mass observed for the CB N330-filled blends, particularly at intermediate SBR contents, suggests increased carbonaceous residue formation and greater resistance to thermal decomposition under the applied test conditions^131^.

## Conclusion

This study investigated the combined effects of NR/SBR blend composition and carbon black grade (N330 and N550) at a fixed filler loading of 50 phr on the mechanical and thermal properties of vulcanized rubber compounds.

- The results demonstrated that both blend composition and carbon black grade significantly influenced tensile strength, tensile modulus, elongation at break, toughness, tear strength, hardness, abrasion resistance, and thermal stability.
- The CB N330-filled blends consistently exhibited higher mechanical performance compared with the corresponding CB N550-filled compounds.
- Equilibrium swelling measurements indicated that the CB N330-filled compounds generally showed higher apparent crosslink density, contributing to their superior mechanical performance, enhanced abrasion resistance, and improved thermal stability.
- The CB N330-filled blends consistently exhibited lower relative volume loss than the corresponding CB N550-filled compounds. Among the investigated formulations, the NR/SBR (75/25) blend reinforced with CB N330 exhibited the highest abrasion resistance and the most balanced combination of strength, stiffness, toughness, hardness, and crack resistance.
- Thermogravimetric analysis confirmed that all compounds underwent single-stage thermal degradation. Increasing the SBR content shifted T₁₀ and Tₘₐₓ toward higher temperatures, indicating improved thermal stability.
- The N330-filled blends exhibited higher residual mass during thermal decomposition, suggesting increased carbonaceous residue formation and greater resistance to thermal degradation.

Overall, the results demonstrate that both carbon black characteristics and blend composition play important roles in determining the mechanical, wear, and thermal performance of NR/SBR compounds. The findings identify CB N330 as the more effective reinforcing filler for NR/SBR-based tire tread compounds owing to its superior reinforcing efficiency, which provides an optimal balance of mechanical strength, abrasion resistance, crack resistance, and thermal stability. These findings provide practical guidance for selecting appropriate carbon black grades and NR/SBR blend compositions for the development of high-performance tire tread compounds.

### Future work

The present study focused on evaluating the effects of SBR content and carbon black grade on the mechanical properties, swelling behavior, thermal stability, and abrasion resistance of NR/SBR blends. To provide a more comprehensive assessment of their suitability for tire tread applications, further investigations are recommended. Future work will include the evaluation of rebound resilience, cure characteristics, and dynamic viscoelastic properties. Additional studies on heat buildup behavior, rolling resistance indicators, Payne effect, and tan δ characteristics will provide further insight into energy dissipation and service performance under cyclic loading conditions. Moreover, morphological characterization and filler dispersion analysis will be performed to better understand the reinforcing mechanisms and filler–rubber interactions governing the overall performance of the blends.

## Data Availability

The datasets used or analyzed during the study available from the corresponding author on reasonable request.
